# The Enhancer of Trithorax and Polycomb Corto Interacts with Cyclin G in *Drosophila*

**DOI:** 10.1371/journal.pone.0001658

**Published:** 2008-02-20

**Authors:** Juliette Salvaing, Anja C. Nagel, Emmanuèle Mouchel-Vielh, Sébastien Bloyer, Dieter Maier, Anette Preiss, Frédérique Peronnet

**Affiliations:** 1 Laboratoire de Biologie du Développement, UMR 7622, Centre National de la Recherche Scientifique (CNRS), Université Pierre et Marie Curie-Paris 6, Paris, France; 2 Institut für Genetik (240), Universität Hohenheim, Stuttgart, Germany; Fred Hutchinson Cancer Research Center, United States of America

## Abstract

**Background:**

*Polycomb* (*PcG*) and *trithorax* (*trxG*) genes encode proteins involved in the maintenance of gene expression patterns, notably *Hox* genes, throughout development. PcG proteins are required for long-term gene repression whereas TrxG proteins are positive regulators that counteract PcG action. PcG and TrxG proteins form large complexes that bind chromatin at overlapping sites called Polycomb and Trithorax Response Elements (PRE/TRE). A third class of proteins, so-called “Enhancers of Trithorax and Polycomb” (ETP), interacts with either complexes, behaving sometimes as repressors and sometimes as activators. The role of ETP proteins is largely unknown.

**Methodology/Principal Findings:**

In a two-hybrid screen, we identified Cyclin G (CycG) as a partner of the *Drosophila* ETP Corto. Inactivation of *CycG* by RNA interference highlights its essential role during development. We show here that Corto and CycG directly interact and bind to each other in embryos and S2 cells. Moreover, CycG is targeted to polytene chromosomes where it co-localizes at multiple sites with Corto and with the PcG factor Polyhomeotic (PH). We observed that *corto* is involved in maintaining *Abd-B* repression outside its normal expression domain in embryos. This could be achieved by association between Corto and CycG since both proteins bind the regulatory element *iab-7* PRE and the promoter of the *Abd-B* gene.

**Conclusions/Significance:**

Our results suggest that CycG could regulate the activity of Corto at chromatin and thus be involved in changing Corto from an Enhancer of TrxG into an Enhancer of PcG.

## Introduction

In *Drosophila*, the Bithorax-complex (BX-C) contains the three *Hox* genes, *Ultrabithorax* (*Ubx*), *abdominal-A* (*abd-A*) and *Abdominal-B* (*Abd-B*), that specify the identities of the third thoracic segment (T3) and the eight abdominal segments (A1 to A8) [1]. These genes are expressed in spatially regulated patterns during embryonic development thanks to maternal, gap and pair-rule proteins. Their large *cis-*regulatory sequences are modular and allow parasegmental regulation. These sequences contain different classes of elements such as initiation elements that respond to early segmentation gene products, insulators and promoter targeting sequences (reviewed in [2]).

*Hox* expression is maintained in the original pattern during later stages of development by the *Polycomb-group* (*PcG*) and *trithorax-group* (*trxG*) genes. In mutants of *PcG* or *trxG* genes, *Hox* patterns are established correctly but are not maintained. PcG proteins keep *Hox* genes silenced whereas TrxG proteins keep *Hox* genes activated thus counteracting PcG action [3], [4]. PcG and TrxG proteins are required for the maintenance of many gene expression patterns [5]. These maintenance proteins form heteromultimeric complexes that bind to chromatin and alter its structure. Current models propose that PcG complexes lead to compact, transcriptionally inactive chromatin, whereas TrxG complexes maintain chromatin in an open conformation that facilitates transcription. In *Drosophila*, several PcG and TrxG complexes have been purified so far: the Polycomb Repressive Complex 1 (PRC1), the Polycomb Repressive Complex 2 (PRC2), the PhoRC complex, the Pcl-PRC2 complex, the Trithorax Activating Complex 1 (TAC1) and the Brahma Complex (BRM) also called SWI/SNF complex. They are extremely large complexes that contain several proteins including chromatin modifying enzymes such as histone methyl-transferases, acetyl-transferases or deacetylases [5]–[8].

Although most *PcG* mutations suppress *trxG* mutations and *vice versa*, a large screen to identify modifiers of the *trxG* gene *ash1* allowed isolation of enhancers that were previously identified as *PcG* [*E(z), E(Pc), Asx, Scm, Psc* and *Su(z)2*] [9]. These genes were then called *Enhancers of Trithorax and Polycomb* (*ETPs*). Further molecular data showed that some *ETPs* encode members of PRC complexes, such as E(Z), PSC or SCM, while some do not. Recently, Grimaud *et al.* proposed to reclassify these maintenance proteins, the label PcG being kept for members of PRC silencing complexes and the label TrxG for members of complexes that counteract PcG-mediated silencing [10]. A third class of proteins would be represented by PcG/TrxG DNA-binding recruiters or specific co-factors. We will keep here the term ETP for those maintenance proteins that play a dual role in PcG and TrxG functions without belonging to any PcG or TrxG complexes identified so far. The GAGA factor, Gaf, encoded by *Trithorax-like* (*Trl)*, falls into this category. Indeed, it was first described as an activator of *Hox* genes, and later shown to play a role in the recruitment of PcG complexes without co-purifying with any PRC silencing complexes [11], [12]. The HMG protein DSP1 also meets the criteria to be an ETP: *dsp1* mutants exhibit *Hox* gene loss-of-function phenotypes but DSP1 is also important for PcG recruitment to chromatin [13], [14]. We have previously shown that *corto* behaves genetically as an *ETP*. *corto* mutants present *PcG* as well as *trxG* phenotypes and enhance the phenotypes of some *PcG, trxG* and *ETP* mutants [15], [16]. Corto directly interacts with Gaf and DSP1 suggesting that ETPs are involved in collaborative processes [16], [17].

PcG, TrxG and ETP proteins bind DNA sequences called PRE/TRE that carry the information for the active or silent state of the gene they control (reviewed in [18]). Some PRE/TRE have been shown to maintain this transcriptional state throughout cellular divisions in absence of the initial activator or repressor [19], [20]. Despite massive efforts towards identification of PcG complex targets at genome scale [21]–[23], the mechanism by which the active or inactive state of PRE/TRE is conserved throughout several cell cycles remains still largely unknown. Many *PcG* and *ETP* mutants [*Asx, corto*, *E(z), Pc, ph*, *Psc, Su(z)2, Trl*] exhibit proliferation defects as well as chromosome condensation and segregation defects. This suggests that maintenance proteins play a general role in cell cycle control [24]–[28]. An attractive hypothesis is that ETPs are critical to maintain the correct association of PcG or TrxG complexes with chromatin during the cell cycle.

In a two-hybrid screen using Corto as bait, we isolated Cyclin G (CycG), the *Drosophila* homologue of the mammalian Cyclin G1 and G2 (CycG1, CycG2). Vertebrate CycG1 is a transcriptional target of the tumor suppressor p53 [29], [30]. It is possibly involved in cell proliferation as it is overexpressed in certain cancer cells [31], [32]. However, CycG1 induces G2/M arrest and cell death in response to DNA damage [33]–[35]. Vertebrate CycG2 acts as a negative regulator of cell cycle, as shown by its high level in cells in which G1/S arrest has been induced by growth inhibitory signals [36], [37].

Here, we address the interactions between Corto and CycG both *in vitro* and *in vivo*. We show that *CycG* plays an essential role during development. Moreover, we show that CycG is targeted to many sites on polytene chromosomes where it co-localizes partially with Corto and with the PcG factor PH. As an ETP, *corto* maintains *Abd-B* repression in embryos. This could be achieved by association between Corto and CycG since both proteins bind to *Abd-B* regulatory elements, including the *iab-7* PRE and the promoter.

## Results

### Drosophila Cyclin G interacts with Corto

**Figure 1:**
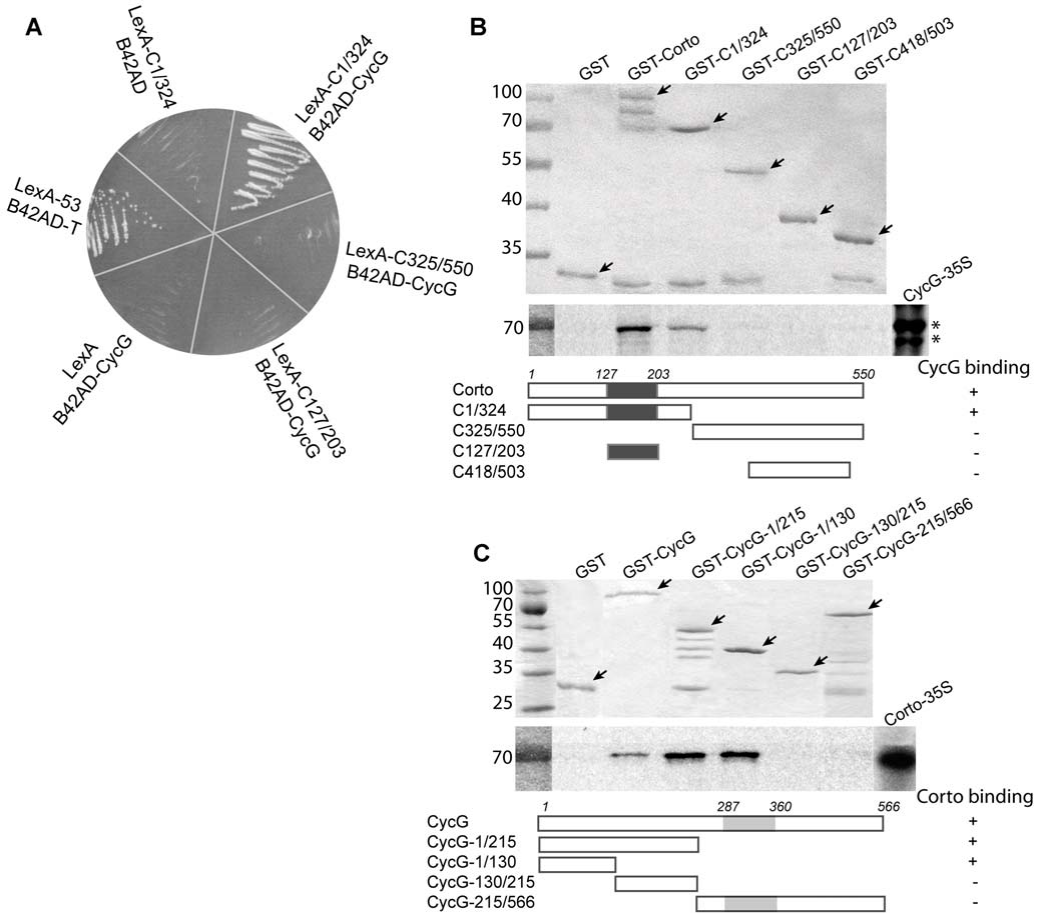
Corto and CycG interact *in vitro*. A. Two-hybrid tests. Three Corto sub-fragments comprising amino-acids 1 to 324 (C1/324), 325 to 550 (C325/550), and 127 to 203 (C127/203) (chromodomain) were fused with LexA DNA binding domain. B42AD-CycG was isolated from an embryonic library using LexA-C1/324 as bait. Interaction was observed only between LexA-C1/324 and B42-CycG. Negative controls: LexA *versus* B42AD-CycG and LexA-C1/324 *versus* B42AD. Positive control: LexA53 (LexA/p53 fusion) *versus* B42AD-T (B42AD/large T-antigen fusion). B. GST pull-down assays. Top: Coomassie staining of fusion proteins (shown by arrows). Middle: Autoradiography. Input (CycG-^35^S) shows two isoforms (asterisks). Bottom: schematic representation of Corto full-length and truncated forms (black box: chromodomain). C. GST pull-down assays. Top: Coomassie staining of the fusion proteins (shown by arrows). Middle: Autoradiography. Input (Corto ^35^S). Bottom: schematic representation of CycG full-length and truncated forms (grey box: cyclin domain).

To further investigate Corto function, we performed a two-hybrid screen for potential Corto partners. As bait, we used the amino-terminal half of Corto containing a chromodomain [16]. A positive clone spanning almost the full-length *CG11525* cDNA (positions 43 to 2263; Accession number NM 079870) encoding Cyclin G (CycG) was isolated. Subsequent two-hybrid assays showed that the chromodomain was not sufficient for interaction with CycG, and that CycG did not interact with the C-terminal half of Corto (figure 1A).

Then, we performed GST pull-down assays. *In vitro* translated CycG protein was retained on GST-Corto beads containing the full-length protein and on GST-C1/324 beads containing the amino-terminal half of Corto, but not on the other GST-Corto fusion proteins tested (figure 1B). Reciprocally, *in vitro* translated Corto protein was retained on GST-CycG beads which contained the full-length CycG protein. Corto was not retained on GST-CycG-215/566 which contains the cyclin domain but was bound by the N-terminal CycG-region (figure 1C). These results corroborate the two-hybrid results and indicate that the amino-terminal half of Corto interacts with the amino-terminal end of CycG.

### CycG is an essential gene in flies

**Figure 2:**
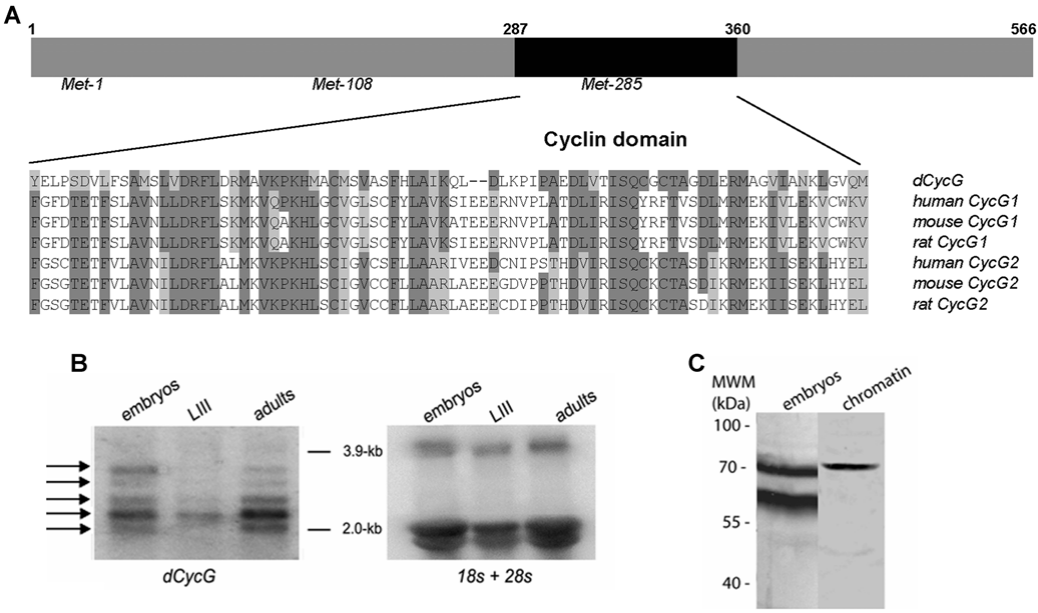
Alignment of cyclin domains of *Drosophila* and mammalian CycG proteins. A. Alignment of the cyclin domains of *Drosophila* CycG (NM 524609), human CycG1 (NP 954854), mouse CycG1 (NP 033961), rat CycG1 (NP 037055), human CycG2 (NM 004345), mouse CycG2 (NP 031661) and rat CycG2 (NP 446451) (dark grey: identical amino-acids, light grey: similar amino-acids). B. Developmental Northern blot. Poly(A^+^) RNA from *w^1118^* embryos, third instar larvae (LIII) and adult flies were probed with ^32^P-labelled *CycG* cDNA (left). Arrows point to five mRNA species ranging from 2.0 to 3.5 kb in size that may correspond to CG11525 RA-RE transcripts predicted in Flybase. The same filter was hybridized with ^32^P-labelled *18S* and *28S* probes (right) for loading control. C. Total protein (left) and chromatin (right) extracts were prepared from *w^1118^* 0–14 h embryos and analysed using rabbit anti-CycG antibodies.

The cyclin domain of *Drosophila* CycG is highly similar to the cyclin domains of vertebrate CycG1 and CycG2 (42% and 46% identity, respectively; figure 2A). In agreement with genome annotations, Northern blot analysis revealed 5 different mRNAs ranging between 2.0 and 3.5 kb. All transcripts were found throughout development although notably less abundant in third instar larvae (figure 2B). Antibodies were raised against the N-terminal part of CycG. Two isoforms of 68 kDa and 60 kDa were revealed in total embryonic extracts whereas only the 68 kDa species was found in chromatin extracts (figure 2C). Three translation start sites are predicted in CycG using ATGpr software [38], resulting in putative proteins with molecular weights of 63, 50 and 30 kDa, respectively (figure 2A). Our antisera do not allow testing for the presumptive 30 kDa isoform. However, the two isoforms we detect probably correspond to the two larger predicted proteins.

**Figure 3:**
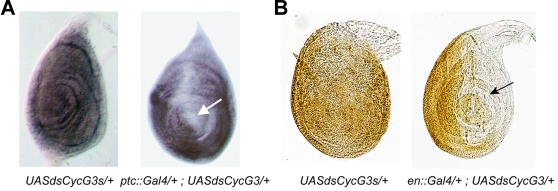
*CycG* is an essential gene in *Drosophila*. A. Validation of *RNAi* efficiency by *in situ* hybridization. Leg imaginal discs from *ptc::Gal4>UAS::dsCycG3* third instar larvae were hybridized using *CycG* as a probe. Expression of the *CycG* mRNA was specifically reduced in the *ptc* expression domain along the antero-posterior border (arrow). B. Validation of *RNAi* efficiency by immuno-labelling. Leg imaginal discs from *en::Gal4>UAS::dsCycG3* were immuno-labelled using rabbit anti-CycG antibodies. The expression of CycG was specifically reduced in the *en* expression domain *i.e.* in the posterior compartment (arrow), whereas it was ubiquitous in the control (*UAS::dsCycG3*).

Since no mutant of *CG11525/CycG* was available, we designed a *P{UAS::dsCycG}* construct to inactivate the gene by RNA-interference (RNAi) as described previously [39]. Tissue specific RNAi using various Gal4 driver lines resulted in a considerable downregulation of *CycG* activity as visualized by reduction of *CycG* mRNA and CycG protein levels (figure 3), respectively. Ubiquitous downregulation of *CycG* (*da::Gal4; UAS::dsCycG* or *Act::Gal4; UAS::dsCycG*) animals led to lethality of late third instar larvae or pharates. However, the percentage of dead animals varied depending on the transgenic line: lethality of *da::Gal4>UAS::dsCycG2* was estimated to 31% in females and 56% in males whereas no *da::Gal4>UAS::dsCycG3* or *Act::Gal4>UAS::dsCycG3* adults were obtained (table 1). Lethality was complete in *Act::Gal4>UAS::dsCycG2* males and reached 86% in *Act::Gal4>UAS::dsCycG2* females. We observed that males of this genotype never undergo metamorphosis and stop their development as third instar larvae, dying after a few days, whereas most females die as late pharates. Thus, these two lines interfere with CycG activity to different degrees, indicative of partial inactivation. Overexpression of *CycG* with an ubiquitous driver (*da::Gal4>UAS::CycG* or *Act::Gal4>UAS::CycG*) suppressed the lethality induced by *UAS::dsCycG3* suggesting that it was linked to specific inactivation of *CycG* and not to Off-target effects (table 1). Taken together, these results show that *CycG* plays an essential role during development.

**Table 1:** Lethality of *RNAi CycG* flies.

Genotype	Lethality observed (%)	Emerged flies
		
*X/X ; UAS::dsCycG2/+ ; da::Gal4/+*	31	230
*X/Y ; UAS::dsCycG2/+ ; da::Gal4/+*	56	185
*X/X ; UAS::dsCycG3/da::Gal4*	100	-
*X/Y ; UAS::dsCycG3/da::Gal4*	100	-
*Act::Gal4/X ; UAS::dsCycG2/+*	86	18
*Act::Gal4/Y ; UAS::dsCycG2/+*	100	-
*Act::Gal4/X ; UAS::dsCycG3/+*	100	-
*Act::Gal4/Y ; UAS::dsCycG3/+*	100	-
*X/X ; UAS::CycG/+ ; da::Gal4/UAS::dsCycG3*	0	145
*X/Y ; UAS::CycG/+ ; da::Gal4/UAS::dsCycG3*	0	153
*Act::Gal4/X ; UAS::CycG/+ ; UAS::dsCycG3/+*	0	168
*Act::Gal4/Y ; UAS::CycG/+ ; UAS::dsCycG3/+*	0	112

### Corto and CycG interact in vivo and co-localize at multiple sites on polytene chromosomes

**Figure 4:**
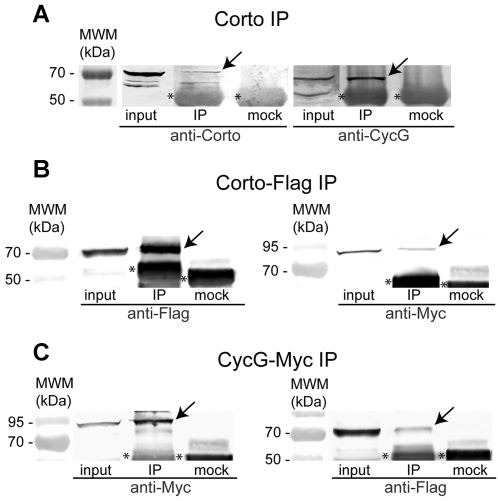
Corto and CycG interact *in vivo.* A. CycG co-immunoprecipitates with Corto in embryonic extracts. Protein extracts from 0–14 h embryos were incubated with rabbit anti-Corto antibodies (IP) or rabbit preimmune serum (mock). Western blot analysis was performed using rat anti-Corto antibodies (left) or rat anti-CycG antibodies (right). Note specific precipitation of Corto (70 kDa) and the 68 kDa CycG species (arrows). The asterisks label unspecific IgG signals in all panels. B. Myc-CycG co-immunoprecipitates with Corto-Flag in S2 cell extracts. S2 cells were co-transfected with *pAct::Corto-Flag* and *pAct::Myc-CycG*. Proteins extracts were incubated with either mouse anti-Flag antibodies (IP) or mouse anti-HA antibodies (mock). Western blot analysis was performed using mouse anti-Flag antibodies (left) or mouse anti-Myc antibodies (right). Specific precipitation of Corto-Flag (left) or Myc-CycG (right) is indicated by arrows. C. Corto-Flag co-immunoprecipitates with Myc-CycG in S2 cell extracts. S2 cells were co-transfected with *pAct::Corto-Flag* and *pAct::Myc-CycG*. For immunoprecipitation we used either mouse anti-Myc antibodies (IP) or mouse anti-HA antibodies (mock), and for detection, mouse anti-Myc antibodies (left) or mouse anti-Flag antibodies (right). Specific precipitation of Myc-CycG (left) or Corto-Flag (right) is indicated by arrows. 4% of the starting material used in each IP (input) and 50% of the immunoprecipitated material were loaded onto the gel in all our assays.

*In vivo* physical interactions between Corto and CycG were first analysed by co-immunoprecipitation of total embryonic protein extracts (figure 4A). Only the 68 kDa and not the 60 kDa CycG isoform co-immunoprecipitated with Corto. We cannot exclude that the latter may be hidden by co-migrating IgG. To confirm this result, we co-transfected Schneider S2 cells with *pAct::Corto-Flag* and *pAct::Myc-CycG* and probed for interaction using anti-Flag and anti-Myc antibodies. Myc-CycG co-immunoprecipitated with Corto-Flag and conversely (figure 4B–C).

**Figure 5:**
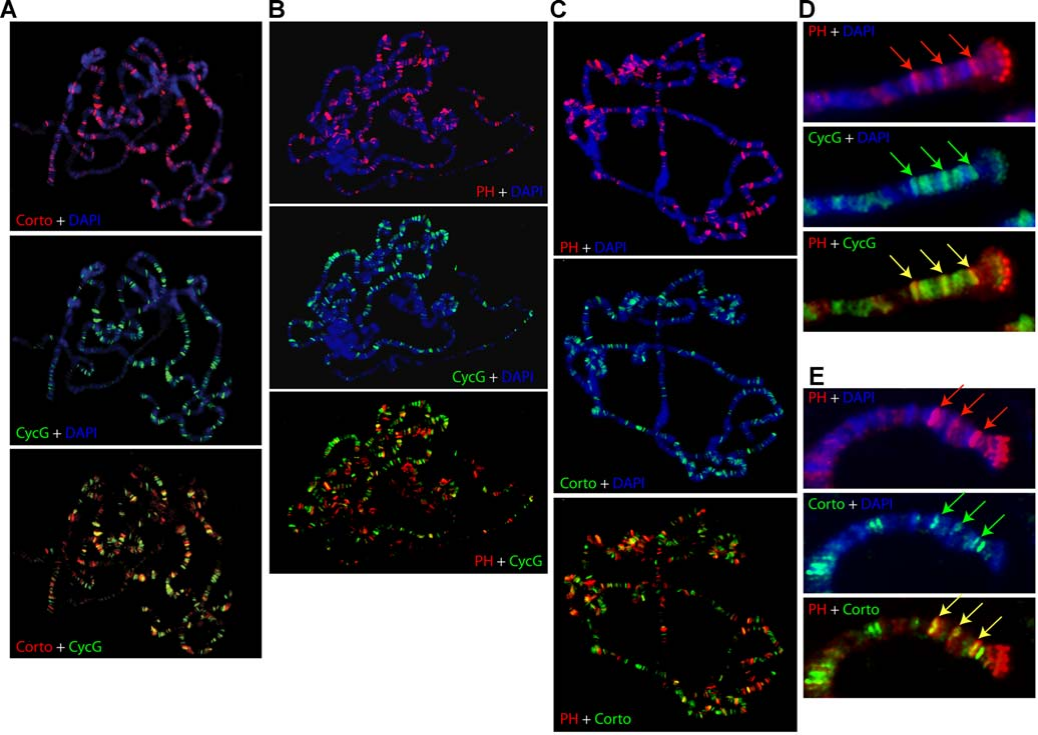
Corto, CycG and PH partially co-localize *in vivo.* Simultaneous detection of Corto (red) and CycG (green) (A), PH (red) and CycG (green) (B), PH (red) and Corto (green) (C) on polytene chromosomes stained with DAPI (blue). Close-ups of chromosome 3 L ends showing 3 loci simultaneously bound by PH (Red) and CycG (green) (D), and the same 3 loci simultaneously bound by PH (red) and Corto (green) (E). Overlaps appear yellow.

Corto was previously shown to bind polytene chromosomes at multiple discrete loci suggesting that it might participate in the regulation of many genes [17]. Using CycG antisera, we first showed that *CycG* is ubiquitously expressed in embryos and larvae (not shown). To test whether interactions between Corto and CycG could take place on chromatin, we explored the binding of CycG to polytene chromosomes (figure 5A). We detected CycG at multiple discrete sites. 30 to 40% of these sites overlapped with Corto binding sites suggesting that Corto and CycG could indeed interact on chromatin.

We next asked whether Corto was essential for CycG recruitment to chromatin, and analysed CycG fixation on polytene chromosomes derived from *corto^07128^/Df(3R)6–7* larvae. On the whole, no modification of the CycG binding pattern was observed (data not shown) suggesting that CycG recruitment does not depend on Corto. We have previously shown that Corto shares many sites on polytene chromosomes with PcG proteins ([16], [17] and figure 5C). We observed that CycG also shares many sites with PH (figure 5B). Moreover, some sites were simultaneously occupied by Corto, CycG and PH, suggesting that the interaction between Corto and CycG could be related to PcG function (figure 5D, E).

### corto participates in the regulation of Abd-B expression in embryos

**Figure 6:**
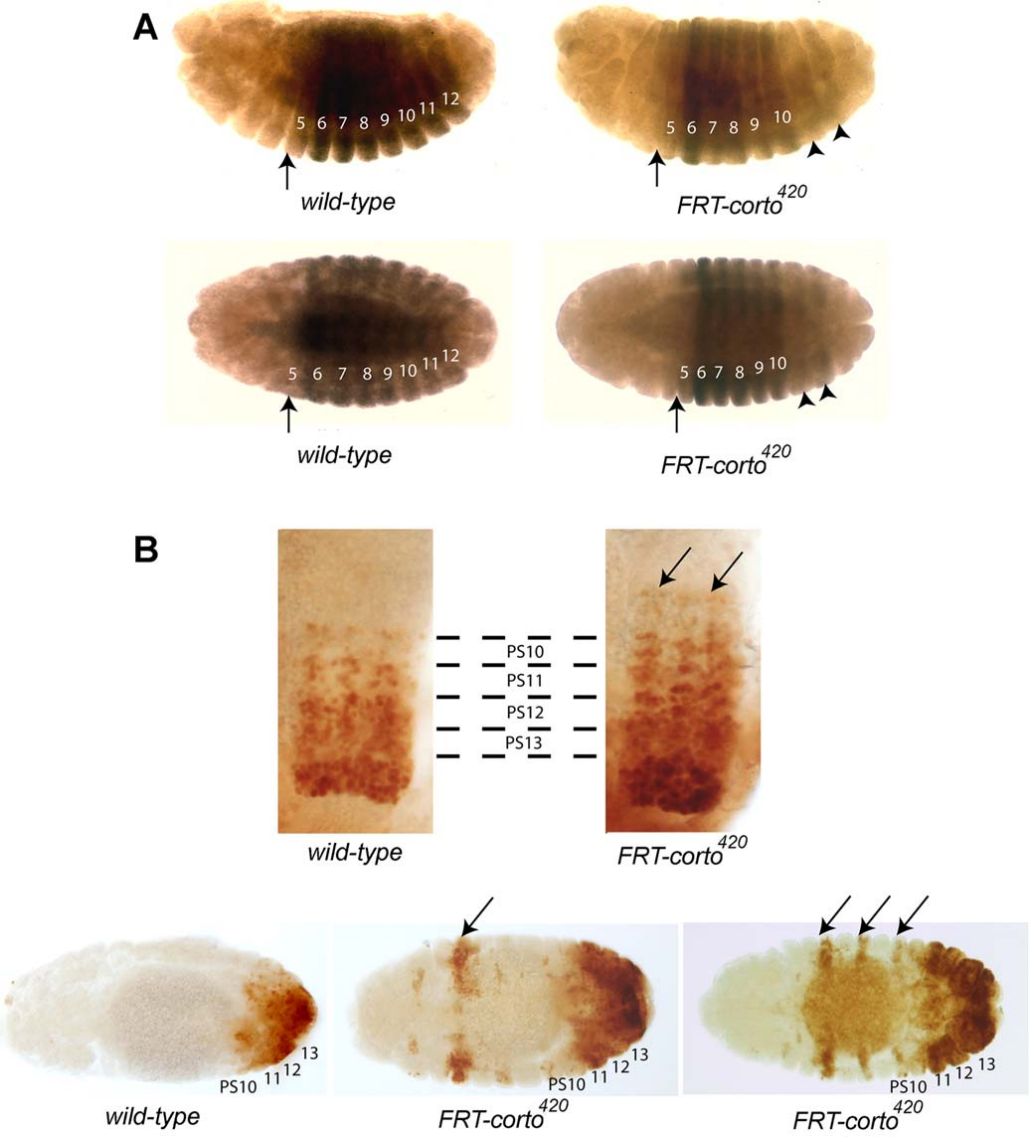
Expression of Hox genes in *corto* mutant embryos. A. Anti-Ubx antibody staining of *w^1118^* embryos, or embryos depleted of maternal and zygotic Corto. Upper row, lateral view and lower row, dorsal view. (B) Dorsal view. In wild-type embryos (left), *Ubx* is expressed from parasegment PS5 to PS12 with a higher expression in PS6. In *corto* embryos (right), the anterior boundary of *Ubx* is not modified but *Ubx* is strongly downregulated in PS11 and PS12 (arrowheads) . Parasegment numbers are indicated in white and the anterior border of *Ubx* expression by arrows. B. Anti-AbdB antibody staining of *w^1118^* embryos, or embryos depleted of maternal and zygotic Corto. Upper row, close-ups of the ventral nerve chord. In wild-type embryos (left), *Abd-B* is expressed in a decreasing gradient from PS13 to PS10. In *corto* embryos (right), this gradient extends anteriorly to PS9 and PS8. Moreover, expression of *Abd-B* seems to be higher in PS13 to PS10. Lower row, dorsal view of whole mount embryos showing ectodermal *Abd-B* expression; note ectopic expression of *Abd-B* in anterior parasegments in *corto* mutant embryos (arrows).

Previous findings indicate that *corto* is involved in the regulation of Hox genes such as *Scr* or *Ubx* in larvae [15], [17]. Interestingly, in *corto* germinal clone embryos, *Ubx* was strongly down-regulated in parasegments (PS) 11–12 whereas normally expressed in more anterior segments ([15] and figure 6A). In wild-type embryos, *Abd-B* expression domain extends from PS 10 to 13 [40], [41]. In light of the posterior prevalence phenomenon, *Ubx* down-regulation could be due to up-regulation of *Abd-B* in the same segments. Indeed, in *corto* germinal clone embryos, the expression of *Abd-B* not only increased in the normal *Abd-B* expression domain but also extended more anteriorly than PS10 (figure 6B). Moreover, discontinuous ectopic expression of *Abd-B* occurred in some anterior parasegments indicative of homeotic transformation towards more posterior identities. These results suggest that *corto* participates in maintenance of *Abd-B* repression in embryos.

### Corto and Cyclin G bind the iab-7 PRE and the promoter of Abd-B

**Figure 7:**
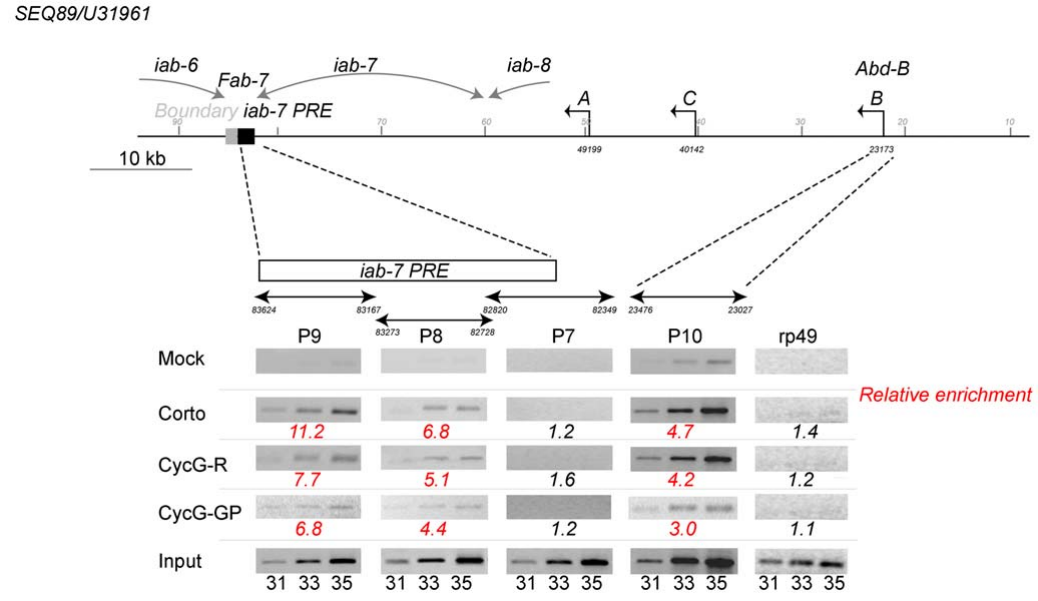
Corto and CycG bind the *iab-7* PRE and the promoter of *Abd-B*. Schematic representation of the *Abd-B* locus (numbers and transcript names refer to SEQ89/U31961) [44]. Corto or CycG XChIP was performed using total chromatin from 0–14 h embryos. Fragments around 500 bp from the *iab-7* PRE (p9, p8) and overlapping the *iab-7* PRE (p7) or the *Abd-B* B promoter (p10) were amplified with specific primers using the immuno-precipitated DNA [42], [45]. A fragment from the *rp49* gene was used as a negative control. The 31^st^, 33^rd^ and 35^th^ PCR cycle samples are shown. Relative enrichment was estimated for the 33^rd^ PCR cycle sample from the ratio between Corto or CycG immunoprecipitations and mock signals from three independent experiments. The input track shows amplification of DNA from total chromatin with the same primers (Mock: rabbit preimmune serum, Corto: rabbit anti-Corto, CycG-R: rabbit anti-CycG, CycG-GP: guinea-pig anti-CycG). The *iab-6*, *iab-7* and *iab-8 cis*-regulatory domains are indicated.

Co-localization of Corto and CycG proteins on polytene chromosomes raises the possibility that CycG and Corto belong to a complex that regulates *Abd-B* expression. To address this possibility, we investigated whether both proteins bind to *Abd-B cis-*regulatory sequences in embryos. We performed immunoprecipitation on formaldehyde cross-linked chromatin (XChIP) from 0–14h embryos. The co-immunoprecipitated DNA was amplified using primer pairs corresponding either to the promoter region of *Abd-B* (generating fragment p10), to the *iab-7* PRE (generating fragments p9, p8 and p7) or to *rp49* as a negative control. We found that Corto and CycG were both present on the promoter and on the *iab-7* PRE (figure 7). As previously described for Polycomb (Pc), we observed prominent binding for the p9 fragment that contains the minimal PRE [42], [43]. These results strongly suggest that Corto and CycG directly regulate *Abd-B* expression.

## Discussion

We have identified Cyclin G as a new binding partner of the ETP Corto in *Drosophila melanogaster*. *CycG* inactivation leads to lethality showing that this gene is essential in flies. Mammalian genomes encode two G-type cyclins, CycG1 and CycG2, the first one being mainly nuclear whereas the second is mainly cytoplasmic [46]. *Drosophila* has a single homologue, however, it produces at least two different protein isoforms, only the larger being associated with chromatin. These isoforms could combine CycG1 and CycG2 functions. In *Drosophila*, large scale two-hybrid screens suggested binding of CycG to various Cyclin-Dependent Kinases (CDK) (Cdc2 and Cdk4) [47], [48]. Corto and CycG interact *in vitro* as well as *in vivo* and form a complex in embryos and presumably also on chromatin. Moreover, Corto interacts with the amino-terminal domain of CycG, which is compatible with the simultaneous binding of CDK and cell-cycle control function of CycG.

Requirement of *PcG*, *trxG* and *ETP* genes in cell-cycle control has already been shown in *Drosophila* [49], [50]. Interestingly, *PcG* and *trxG* genes are also involved in self-renewal and proliferation of hematopoietic stem cells in vertebrates [51], [52]. One way they might control cell proliferation is by an epigenetic regulation of genes involved in cell cycle and cell proliferation. Indeed, homologues of Drosophila E(z) and Brm participate in the transcriptional regulation of *Cyclin A* and *E* in vertebrates, and in *Drosophila*, *Cyclin A* is a PcG target [53]–[55]. Alternatively, PcG, TrxG or ETP proteins may interact directly with cell cycle regulatory proteins. Indeed, it has been shown that Brm interacts with Cyclin E, that Mel-18, a human homologue of Posterior Sex Combs, interacts with Cyclin D2 possibly blocking its interaction with Cdks [56], [57] and we show here that the ETP Corto interacts with CycG. These interactions reveal a potential role for these maintenance proteins in regulating the cell cycle independently of transcriptional regulation. This could be a widespread mechanism by which PcG, TrxG and ETP coordinate the chromatin activity status.

CycG and Corto co-localize on many sites on polytene chromosomes suggesting that they may have regulated associations. Our data show that Corto represses *Abd-B* in embryos and although we were not able to test the role of *CycG* in regulating *Abd-B* expression in embryos, we observed that both Corto and CycG bind the *iab-7* PRE and the promoter of *Abd-B* suggesting that they could cooperate in this function. Nevertheless, neither Corto nor CycG were detected on the BX-C locus in salivary glands suggesting that they regulate *Abd-B* in a tissue-specific manner. The role of the CycG-Corto interaction needs to be further investigated. CycG could regulate Corto activity directly on chromatin by recruiting other factors like kinases or phosphatases thus modifying the phosphorylation status of Corto itself, of histones or other proteins at PRE/TRE and promoters. It has been shown that binding of the PcG protein Bmi1 to chromatin correlates with its phosphorylation status [58], [59]. It will be interesting to investigate whether Corto and CycG bind the *iab-7* PRE and promoter of *Abd-B* simultaneously, to examine their phosphorylation status when bound to chromatin, and to determine if their presence correlates with *Abd-B* transcriptional activity. One interesting possibility would be that CycG is involved in changing Corto from an Enhancer of TrxG into an Enhancer of PcG.

## Materials and Methods

### Drosophila strains and genetics

Details on *Drosophila* strains can be found in *Flybase* [60]. *corto^420^* and *corto^07128^* are strong hypomorphic alleles; *corto^420^/TM6B* was a gift from Roland Rosset [27]. *corto*-deficient germline clones were obtained as previously described [15]. Other strains were obtained from either Bloomington or Kyoto *Drosophila* stock centers. *CycG* transgenic lines were established by standard P-element mediated transformation.

### Plasmid constructs

Corto-GST fusions and *pBS-Corto* were previously described [16]. The *pJG-CycG* plasmid served for PCR amplification of full-length or truncated forms of *CycG* that were subsequently cloned into *pGEX4T-1*. Full length cDNAs were subcloned into pENTR/D-TOPO® and transferred into Gateway™ vectors: *corto* into *pAWF* to obtain *pAct::Corto-Flag*, and *CycG* into *pAMW* to obtain *pAct::Myc-CycG* [61].

*P{UAS::CycG}* was constructed by cloning the entire cDNA as *EcoRI/XhoI* fragment into *pUAST* [62]. *UAS::dsCycG* was constructed as outlined before [39]. About 600 nucleotide coding sequence (codons 72 to 268) was chosen to prepare the RNAi-construct. This segment shows only limited identity to other *Drosophila* genes and none of them conforms to an optimal siRNA. Two possible “Off-Targets” were found, LvpL encoding a larval protein with a predicted role in glucose metabolism and CG15639 encoding an unknown product. In both cases, 21 nucleotides are identical with a GC content of 55–58% instead of the optimal 43–53% [63]. Cloning details are available upon request.

### Antibodies

Corto and Polyhomeotic (PH) antibodies were used as described previously [17]. Antibodies against Abd-B (clone 1A2E9) were obtained from the Developmental Studies Hybridoma Bank. Polyclonal antibodies against CycG were raised against the N-terminal 276 amino-acids of CycG fused to maltose binding protein in rabbit, rat and guinea pig. Their specificity was checked on CycG protein generated by *in vitro* transcription/translation. Monoclonal anti-Flag M2 and anti-HA was from Sigma (F-3165, H-3663) and anti-Myc clone 9E10 from Santa-Cruz Biotechnology.

### Histology

Antibody staining of embryos and larvae was performed using rabbit anti-CycG (1∶40) or mouse monoclonal anti-Abd-B (1∶10) [64]. Co-immunostaining of polytene chromosomes was performed with rabbit anti-Corto (1∶40) and guinea-pig anti-CycG (1∶40) according to [16]. Secondary antibodies (Alexa Fluor® 594 goat anti-rabbit IgG and Alexa Fluor® 488 goat anti-guinea pig; Molecular Probes) were used at a 1∶1000 dilution.

### Protein-protein interactions

A two-hybrid screen was performed using *pEG-C1/324* that encodes amino-acids 1 to 324 of Corto as bait [16]. The embryonic RFLY1 library [65] was transformed into EGY48SHΔSpe [*MATα his3, trp1, ura3, LexAop(x6)-LEU2*] containing the bait. *In vitro* transcription/translation and GST pull-down assays were performed as described [16].

For co-immunoprecipitations, 1 g of 0–14 h *w^1118^* embryos were homogenized in RIPA buffer (50 mM Tris pH 7.5, 150 mM NaCl, 0.5% NP40, 0.1% SDS, 1 mM PMSF) with protease inhibitors (Roche Diagnostics). 500 µl of total extract (about 1 mg) were pre-cleared with protein A plus protein G agarose beads (for polyclonal antibody IP) or protein G beads (for monoclonal anti-Myc, anti-Flag or anti-HA). Input was 20 µl of this mixture. Incubation was with 10 µl of either rabbit pre-immune or Corto antiserum, mouse anti-Flag, anti-Myc or anti-HA overnight at 4°C. The appropriate beads were added and further incubated for 2 h at 4°C. The supernatant was kept; the beads were washed five times with RIPA buffer and finally resuspended in 40 µl of Laemmli buffer. 20 µl of input (4%), 20 µl of supernatant and half of the beads (20 µl) were loaded. Immunoprecipitates were detected with respective antisera developed in rat. *Drosophila* S2 cells were cultivated at 25°C in Schneider medium supplemented with 10% fetal calf serum and antibiotics. Cells were transfected using Effecten® transfection reagent according to the manufacturer (Qiagen). Commonly, 2×10^6^ cells were transfected with 1 µg of each DNA. Cells were collected after 48 h of incubation and homogenized in 500 µl of RIPA buffer.

### Immunoprecipitation of crosslinked chromatin (XChIP)

Chromatin from 0–14 h embryos was formaldehyde cross-linked and immunoprecipitated as described [66] using rabbit anti-Corto (1∶20), rabbit anti-CycG (1∶20), guinea-pig anti-CycG (1:20) or rabbit pre-immune sera (mock) (1∶20). Three independent immunoprecipitations were performed and further analysed. The precipitated DNA was dissolved in 100 µl of TE [10 mM Tris (pH 8.0), 1 mM EDTA] and 1 µl was used per PCR reaction. Three primer pairs spanning the *iab-7* PRE (p7, p8, p9) and one primer pair from the promoter region of *Abd-B* (p10) were used [42], [45]. *rp49* was used as negative control (primers 5′ CCC AAG ATC GTG AAG AAG CG 3′ and 5′ AGA TAC TGT CCC TTG AAG CG 3′). PCR schemes were as follows: 94°C for 3 minutes; 94°C for one minute, 45°C (p7, p9), 50°C (p8, p10, rp49) for one minute, 72°C for one minute, 36 cycles; 72°C for 10 minutes. 5 µl samples were taken every 2 cycles from the 29^th^ to the 35^th^ cycle to determine the linear range of amplification. PCR products were quantified using ImageJ and results of three independent experiments were normalized against the mock immunoprecipitation.
